# Application of ε‐polylysine in extending the storage period of pork jerky

**DOI:** 10.1002/fsn3.2289

**Published:** 2021-05-04

**Authors:** Yizhuo Zhang, Changqing Zhao, Xingxiu Zhao, Yiguo He

**Affiliations:** ^1^ College of Bioengineering Sichuan University of Science and Engineering Yibin China

**Keywords:** microorganism, pork jerky, preservative, storage period, ε‐polylysine

## Abstract

In this experiment, natural nontoxic preservative ε‐polylysine (ε‐PL) was used as a natural preservative in pork jerky. The pork jerky samples with ε‐PL (experimental group) and without ε‐PL (blank group) were stored at the 27 and 37℃. Then, the number of microorganisms, total volatile basic nitrogen (TVB‐N), pH, and water activity (Aw) of each group were tested to test the antiseptic effect of ε‐PL. The results showed that due to the *Staphylococcus aureus* was detected, the storage period of the blank group at 27 and 37°C was 15 and 9 days, respectively. However, *Coliforms*, *Staphylococcus aureus*, *Salmonella*, and *Shigella* were not detected in the experimental group on the 60th day. The experimental group all accord with the national standard for the quantity of microorganisms in meat jerky. The TVB‐N content of the blank group reached 14.00 mg/100 g (15th day, 27°C) and 14.93 mg/100 g (9th day, 37°C) at the end of the storage period, while the TVB‐N content of the experimental group was 11.20 mg/100 g (60th day, 27℃) and 15.86 mg/100 g (60th day, 37℃), and the increase rate of TVB‐N in the blank group was greater than the experimental group, indicating that ε‐PL can play a better microbial stabilization effect in pork jerky. The test of pH and Aw showed that ε‐PL can stabilize the quality of pork jerky. Finally, the antiseptic effect of ε‐PL was comparable to many chemical preservatives. This experiment confirms that ε‐PL played an important role in extending the storage period of pork jerky.

## Graphical abstract

ε‐Polylysine has excellent antimicrobial activity and quality stability. ε‐Polylysine can extend the storage period of pork jerky to 180 days. ε‐Polylysine has a high research value in meat jerky and other products.

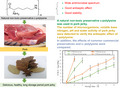

## 1 INTRODUCTION

As one of the most important animal foods on the table, pork is not only of high nutritional value, but also easy to be digested and absorbed. With the continuous improvement of the quality of life, people have higher requirements for the quality of pork, especially the rich nutrition, attractive color, and storable pork products are more attractive to consumers. Therefore, pork is processed into fast meat products such as sausages, pork floss, and pork jerky. Pork jerky is a kind of meat jerky product that is mainly processed from lean pork through slicing, cooking, roasting, cooling, packaging, and sterilization. Pork jerky has unique flavor and easy to eat, which is attract consumers (Zhao et al., 2016).

Pork jerky is rich in nutrients, which creates a good condition for the growth and reproduction of microorganisms. The spoilage caused by microorganisms leads to great changes in the quality of pork jerky during the storage period, which affects the storage period of pork jerky. In order to facilitate the preservation and transportation of pork jerky products, it is necessary to adopt appropriate methods to ensure the quality of pork jerky. The traditional methods to extend the storage period of pork jerky include preservation of low‐temperature, preservation of vacuum packaging, preservation of modified atmosphere packaging, and preservative preservation. These preservation methods greatly meet the needs of consumers for pork jerky, but these methods also have limitations (Yong et al., 2017). For example, the method of preservation of vacuum packaging cannot fully guarantee the quality requirements of pork jerky. Lu et al. found that vacuum packaging has a greater impact on the color difference before and after the meat preservation (Lu et al., 2016). Although the preservation of modified atmosphere packaging can effectively inhibit the growth and reproduction of microorganisms, the cost is relatively high. The preservation method of adding chemical preservatives is the most commonly used method; however, due to people's constant pursuit of healthy ecological food, natural preservatives become a research hotspot. Therefore, this experiment aims to select an appropriate natural preservative to ensure the quality of pork jerky and extend the storage period of pork jerky.

ε‐Polylysine (ε‐PL) is a lysine polymer which is produced by microbial metabolism, and ε‐PL is a natural nontoxic preservative that can be used in the food industry. It has good water solubility, heat stability, and can inhibit most gram‐positive and gram‐negative bacteria, molds, and other microorganisms. Furthermore, ε‐PL can be broken down into L‐lysine in the human body, which thus enhances the nutritional value of food. Literature review shows that ε‐PL has a wide range of applications in pork and pork products. Zhang prepared a variety of concentrations of ε‐PL solution for use in preservation of fresh pork and found that the 1.25% and 1.50% concentration of ε‐PL had better antiseptic effect and had inhibitory effects on both gram‐positive and gram‐negative bacteria, which thus extended the storage period of pork (Zhang, 2013). Liu et al. prepared nanoemulsions using volatile oil of star anise, ε‐PL, and Nisin as the main raw materials, which was used it in the preservation of meat, and found that the storage period of meat was extended from 8 to 16 days (Liu et al., 2020). Cui et al. used ε‐PL and polyglutamic acid to synthesize nanoparticles and prepared a new type of packaging material, which found that this packaging material can effectively control the growth of *Staphylococcus aureus* in pork, and it has no bad effect on pork quality (Cui et al., 2018). Yang et al. soaked raw pork in ε‐PL and stored it at 4°C, and the experimental results showed that ε‐PL can inhibit the bacteria growth of raw pork and extend the storage period of raw pork through monitoring the spoilage of raw pork by measuring pH and total volatile basic nitrogen (TVB‐N; Yang et al., 2018).

However, no other published reports were found on pork jerky produced with ε‐PL. In order to evaluate the effect of ε‐PL as a natural preservative for extending the storage period of pork jerky, this experiment added ε‐PL to the production of pork jerky, and a 60‐day microbiological test (*Coliform*, the aerobic plate count, *Staphylococcus aureus*, *Salmonella*, and *Shigella*) and the determination of TVB‐N content were carried out to detect the inhibitory ability of ε‐PL against microorganisms in pork jerky. In addition, the pH and water activity (Aw) of the pork jerky were measured to detect the stabilizing effect of ε‐PL on the quality of pork jerky. Finally, at the 180th day, the microbiological test was carried out on the pork jerky, and at the same time, the preservative effect of different kinds of common preservatives for meat jerky on the market was summarized and compared with the preservative effect of ε‐PL on pork jerky in this experiment on the 180th day. This experiment provides references for researchers to use ε‐PL to extend the storage period of other meat products such as meat jerky.

## 2 EXPERIMENTAL PROCEDURE

### 2.1 Materials

Lean pork, salt, cumin, white sugar, spices, soy sauce, ginger, rapeseed oil, and maltose commercially available were purchased from local market. While ε‐PL (99% purity solid, Jinan Dongxuan Bioengineering Co., Ltd.) and sodium lactate (60% purity liquid, Zhengzhou Boyan Biotechnology Co., Ltd.) used were of food‐grade quality.

### 2.2 Preparation of pork jerky

#### 2.2.1 The pretreatment of raw pork

Lean pork pieces with similar shapes and sizes were selected, rinsed with running water. Then, they were soaked in clean water and cut with a slicer (SS‐85 model, Yongkang Woqu Industry and Trade Co., Ltd.) into sliced meat (length * width * thickness of 6.0 cm * 4.0 cm * 0.4 cm).

#### 2.2.2 Precooking

1,500 ml tap water and 2 g cumin were added into the pot, and 1,000 g sliced pork was added after the water boiled. The precooking was finished when the pork was white, and the sliced pork was drained.

#### 2.2.3 Cooking

The sediment in the precooking broth was discarded, and the sliced pork was put into the broth again. Then, 1 packet of compound seasoning, 120 g white sugar, 40 g salt, 10 g ginger, 60 g edible oil, 18 ml sodium lactate (60%), and 20 g maltose were put into the broth. After cooking for 5 min under 210℃ conditions and cooking for 5 min under 130℃ conditions, 20 g soy sauce was added and all the mixture was cooked for 40 min under 100℃.

#### 2.2.4 Adding ε‐PL and baking

After cooking, the pork slices were soaked in 0.25 g/L ε‐PL solution (Standard for the use of food additives, 2014) for 10 min. Then, the pork slices were placed on the oil‐absorbing paper (Ultraviolet [UV] sterilization), then in the oven (GZX‐9146MBE model, Shanghai Boxun Industrial Co., Ltd.) were baked at 85°C for 90 min to prepare pork jerky as the experimental group. In addition, the pork jerky without ε‐PL was baked in the same steps that was as the blank group.

#### 2.2.5 Weighing, packaging, and sterilization

Under aseptic conditions, the pork jerky was divided into 7 cm*10 cm transparent packaging bags (18 silk). After the bags were sealed (about 4–6 g per bag), each side of the packaged pork jerky was sterilized for 20 min through a 22.5 W UV lamp (Sterilization of food by UV). Finally, the pork jerky samples were divided into two equal parts and stored at 27 and 37°C for detection of microbiological test, TVB‐N, pH, and Aw.

### 2.3 Determination of the storage period of pork jerky

#### 2.3.1 Microbiological test

*Coliform*, the aerobic plate count, *Staphylococcus aureus*, *Salmonella*, and *Shigella* were detected as the microbiological test of this experiment. The detection methods of these microorganisms were according to Chinese national standard (National food safety standard food microbiological‐the Shigella test for detection, 2012; National food safety standard food microbiological‐the *Coliforms* count for determination, 2016; National food safety standard food microbiological‐the aerobic plate count for determination, 2016; National food safety standard food microbiological‐the *Staphylococcus* *aureus* test for detection, 2016 & National food safety standard food microbiological‐the *Salmonella* test for detection, 2016).

#### 2.3.2 Determination of TVB‐N in pork jerky

The determination of TVB‐N in pork jerky was according to national stamdard (National food safety standard‐determination of the total volatile basic nitrogen in food, 2016).

#### 2.3.3 Determination of the pH in pork jerky

5g sample of pork jerky was minced and put it into an Erlenmeyer flask which containing 45 ml distilled water. After stirred evenly, the mixture was filtrated when standing for 30 min. Then, the pH of the filtrate was measured by a precision pH meter (pHS‐25 model, Chengdu Century Ark Technology Co., Ltd.).

#### 2.3.4 Determination of the Aw in pork jerky

The determination of Aw was according to national standard (National food safety standard‐determination of the water activity in food, 2016).

### 2.4 Statistical analysis of data

In this experiment, all experimental data were measured by the average of the three results, and all experimental data were analyzed using SPSS v. 24.0 (SPSS Inc.).

## 3 RESULTS AND DISCUSSION

### 3.1 Microbiological test of pork jerky

According to the requirements of Chinese national standard "GB4789.1‐2016 Food microbiological inspection‐general principles," the aerobic plate count, *Coliforms*, *Staphylococcus aureus, Salmonella*, and *Shigella* should be tested. Table 1 showed the changes in the aerobic plate count of pork jerky with ε‐PL (experimental group) and pork jerky without ε‐PL (blank group) during the storage period.

**TABLE 1:** Changes in the aerobic plate count during the storage of pork jerky (CFU/g)

Storage time (days)	Storage temperature 27℃		Storage temperature 37℃	
	The experimental group	The blank group	The experimental group	The blank group
1	—	—	—	—
3	—	500 ± 50	—	700 ± 150
6	—	800 ± 130	20 ± 3	1,900 ± 60
9	10 ± 2	1,300 ± 30	10 ± 6	3,300 ± 180
12	10 ± 2	1,700 ± 70	40 ± 2	Due to the detection of *Staphylococcus* *aureus*, the end of the storage period was reached, and subsequent test was stopped
15	10 ± 4	2,100 ± 190	60 ± 12	
18	20 ± 5	Due to the detection of *Staphylococcus* *aureus*, the end of the storage period was reached, and subsequent test was stopped	70 ± 7	
21	20 ± 8		50 ± 10	
24	20 ± 7		80 ± 5	
27	40 ± 10		80 ± 6	
30	30 ± 8		50 ± 21	
33	50 ± 6		110 ± 30	
36	40 ± 12		140 ± 15	
39	50 ± 15		200 ± 37	
42	70 ± 10		210 ± 23	
45	80 ± 5		210 ± 5	
48	120 ± 15		220 ± 14	
51	110 ± 20		330 ± 11	
54	150 ± 24		230 ± 50	
57	130 ± 15		340 ± 8	
60	160 ± 6		350 ± 17	

Note

"—" means not detected.

During the 60‐day storage period, *Staphylococcus aureus* was detected in the blank group (27°C) on the 18th day, this meant that the storage period was 15 days, and the aerobic plate count was 2,100 CFU/g when 15 days. In addition, *Staphylococcus aureus* was detected in the blank group (37°C) on the 12th day, this meant that the storage period was 9 days, and the aerobic plate count was 3,300 CFU/g when 9 days. And *Coliforms*, *Salmonella,* and *Shigella* were not detected in all blank groups. On the 60th day, the aerobic plate count in pork jerky with ε‐PL was 120 CFU/g (27°C) and 150 CFU/g (37°C), respectively. And *Coliforms*, *Staphylococcus aureus*, *Salmonella*, and *Shigella* were all not detected in all experimental groups. The test results of the experimental groups met the national standard "GB2726‐2016 National food safety standard‐cooked meat products," which meant the aerobic plate count in meat jerky was less than 10,000 CFU/g, the *Coliforms* group was less than 10 CFU/g, and *Staphylococcus aureus*, *Salmonella*, and *Shigella* were not detected.

This was because ε‐PL has a broad antibacterial spectrum and has an inhibitory effect on gram‐positive and gram‐negative bacteria. The bacteriostatic mechanism of ε‐PL was that it acted on the cells of microorganisms and effected on the cell membrane system, enzymes, proteins, or genetic material to destroy the cell structure of microorganisms, making the microorganisms lose normal physiological functions that cause cell death, which thereby achieved the purpose of inhibiting the growth and reproduction of microorganisms (Liu, 2016; Shao et al., 2020). In addition, because ε‐PL was easily soluble in water, the surface of the sliced pork after prebaking for 30 min was dry and could achieve a good absorption effect after soaking in ε‐PL solution. At the same time, because ε‐PL plays a better preservative effect on neutral and weakly alkaline food, and the pH of pork jerky was 6.0–7.0, it has a good extend effect of storage period on pork jerky.

### 3.2 Determination of the TVB‐N in pork jerky

**FIGURE 1:**
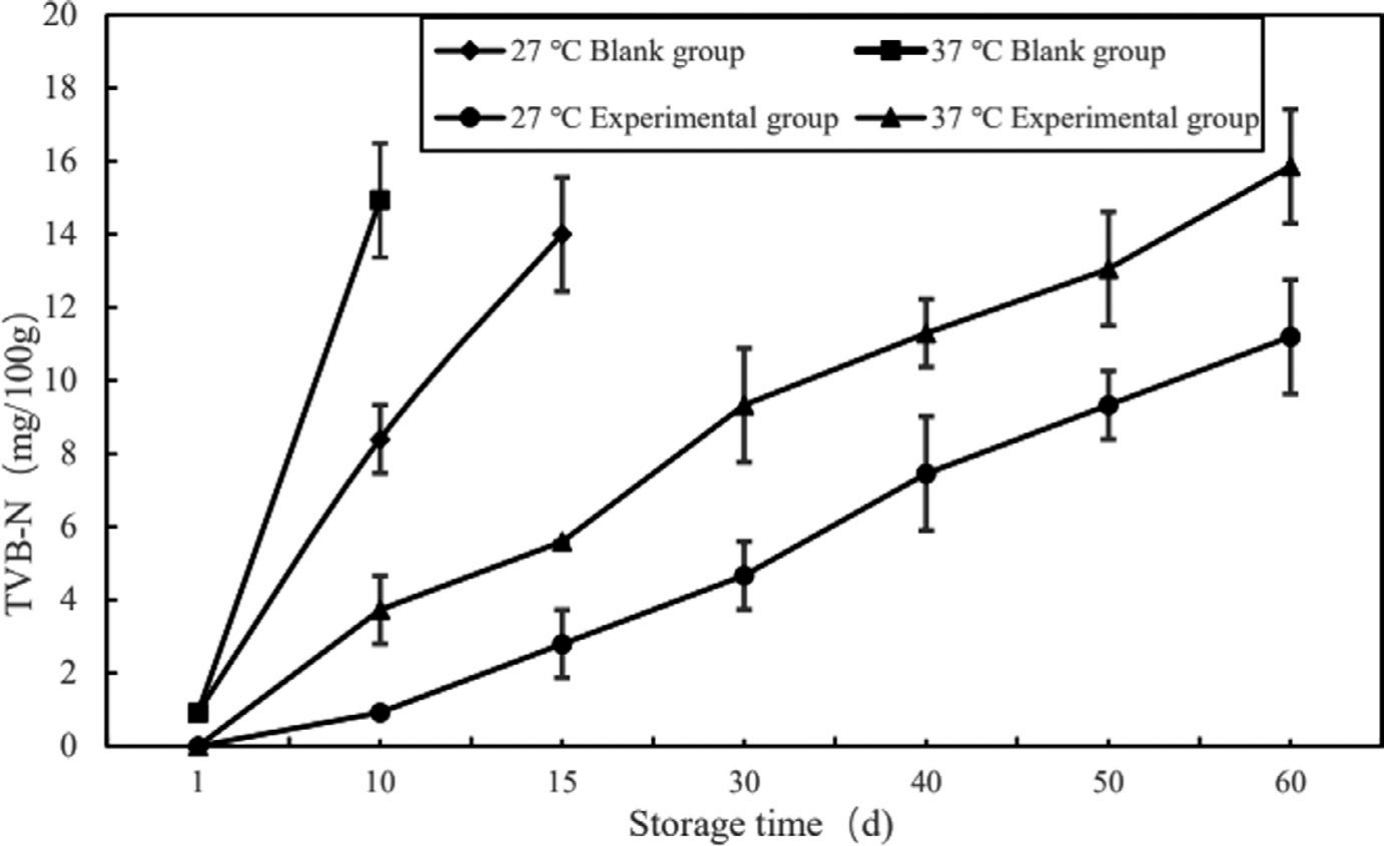
Changes of TVB‐N content in each group of pork jerky during storage period

TVB‐N refers to the decomposition of protein in food under the action of microorganisms to produce ammonia or amines and other volatile nitrogenous substances. The higher TVB‐N content means the higher degree of food spoilage, so TVB‐N can be used to judge the freshness of animal food (Gao, 2019). The changes of TVB‐N content during the storage period of each group of pork jerky were shown in Figure 1.

It can be seen from Figure 1 that the TVB‐N content of the blank group reached 14.00 mg/100 g (15th day, 27℃) and 14.93 mg/100 g (9th day, 37℃) at the end of the storage period, while the TVB‐N content of the experimental group was 11.20 mg/100 g (60th day, 27℃) and 15.86 mg/100 g (60th day, 37℃). The TVB‐N content in the blank group increased rapidly, which was because the aerobic plate count of pork jerky in the blank group increased significantly. And the rapid propagation of microorganisms led to a large amount of nutrients being consumed in the meat jerky; thus, the TVB‐N content increased rapidly. Although the TVB‐N content of pork jerky in the experimental group continued to increase with the storage process, the rate of increase was small. The TVB‐N content of pork jerky stored at 27°C is lower than 37°C (*p* < 0.05), because the storage conditions at 37°C would promote the growth of spoilage microorganisms and accelerate the spoilage rate of pork jerky (Guo et al., 2020). At the same time, it can be seen that ε‐PL as a natural preservative can exert a good antiseptic effect on pork jerky, inhibit the growth of microorganisms in pork jerky effectively, and delay the decay of pork jerky in the experimental group.

### 3.3 Determination of the pH of pork jerky

**FIGURE 2:**
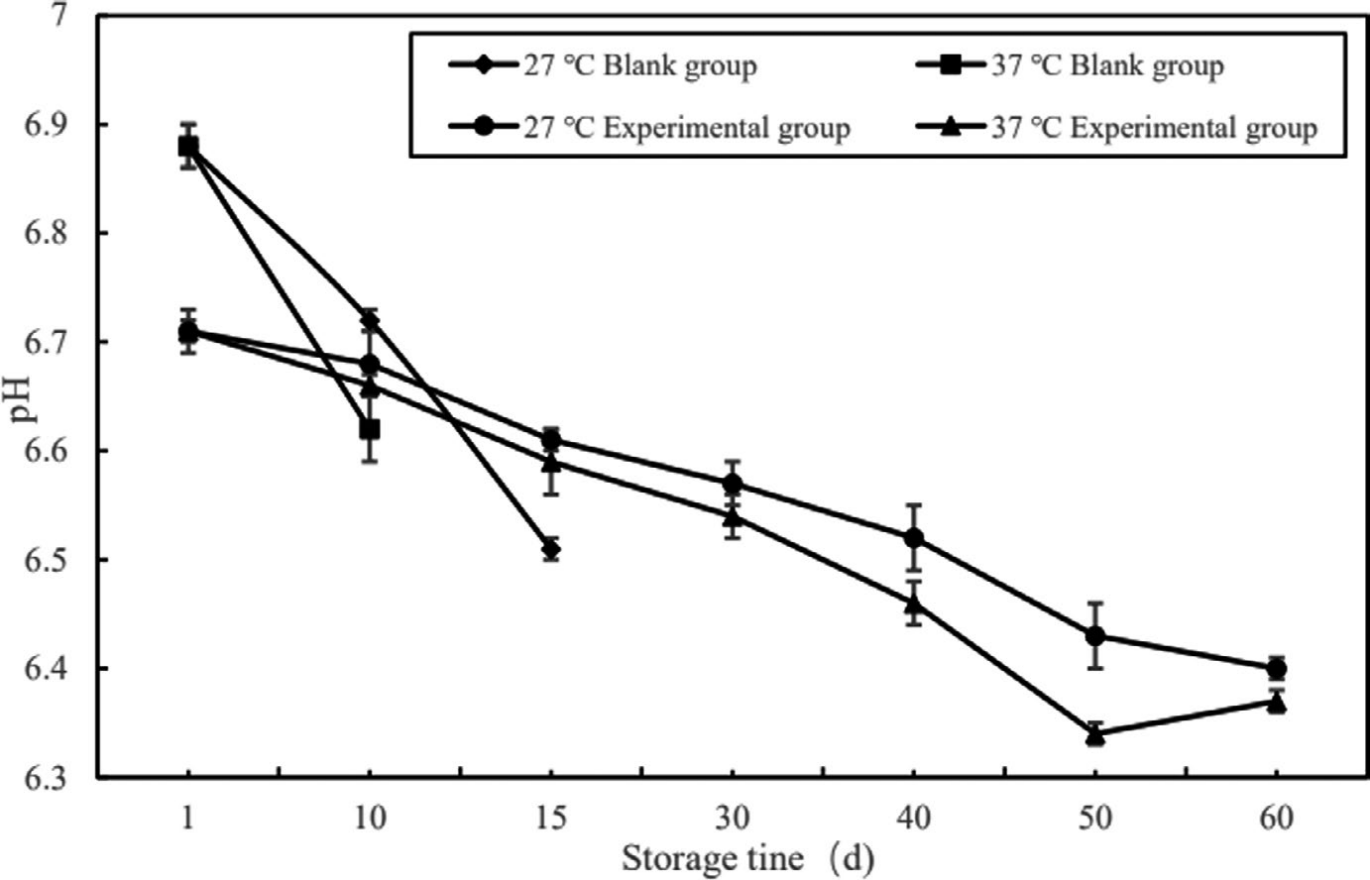
Changes in pH of each group of pork jerky during storage period

The pH of meat jerky not only affects the flavor, but also affects the preservation characteristics of the product, which was an important indicator for judging product quality (Gao, 2019). The determination results of the experimental group and the blank group of pork jerky at different storage temperatures were shown in Figure 2.

It can be seen from Figure 2 that the pH of the pork jerky in the experimental group and the blank group changed to different degrees during the preservation process. The pH of the blank group (27°C) decreased from 6.88 to 6.51 on the 15th day, and the pH of the blank group (37°C) decreased from 6.88 to 6.62 on the 9th day. All blank groups reached the end of the storage period due to the detection of *Staphylococcus aureus*, so the subsequent tests were stopped. On the 60th day, the pH of experimental group (27°C) decreased from the 6.71 to 6.4, and the experimental group (37°C) decreased from the 6.71 to 6.37. The pH decrease rate of the blank group was higher than the experimental group. This was mainly due to the additives in the processing of pork jerky, and the fermentation microorganisms constantly produced lactic acid and other substances reducing pH which lowered the pH of pork jerky (Holmer et al., 2009; Juneja et al., 2016), and thus, ε‐PL could play a good role in inhibiting the growth of microorganisms, making the quality of pork jerky with ε‐PL more stable.

### 3.4 Determination of the Aw in pork jerky

**FIGURE 3:**
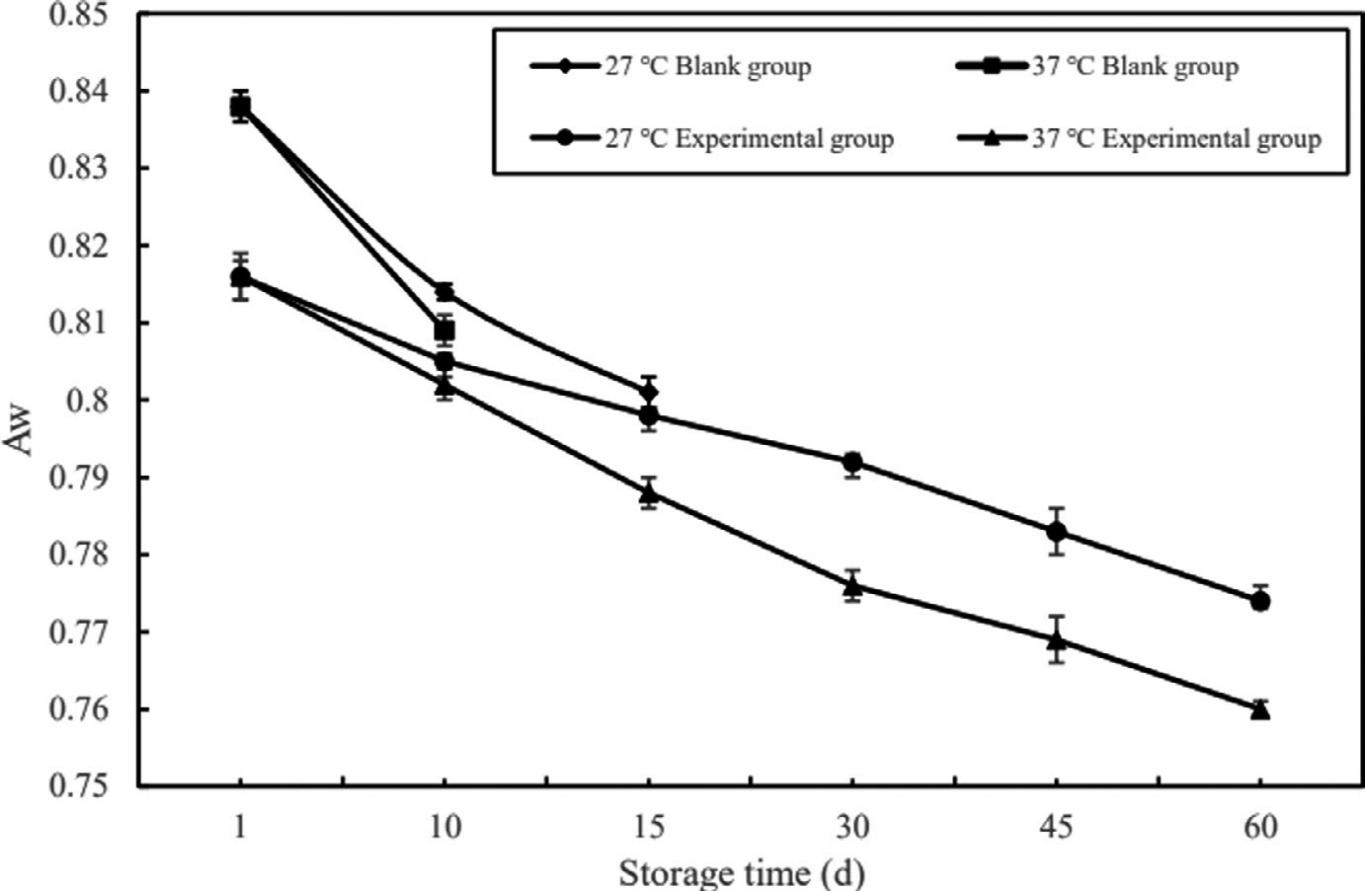
Changes in A_W_ of each group of pork jerky during storage period

Aw represents the combined degree of food and water in the food, and Aw was directly related to the storage period of the food. The changes of Aw during the storage period of each group of pork jerky were shown in Figure 3.

It can be seen from Figure 3 that the Aw of each group of pork jerky decreased slightly with the extension of the storage period under different storage temperatures. In this experiment, sugar, salt, sodium lactate, and other raw materials that can reduced Aw were added, so that the pork jerky had a lower Aw. During the entire storage process, the Aw of pork jerky (37°C) decreased more than pork jerky (27°C). This maybe because the storage conditions at 37°C would promote the growth of spoilage microorganisms and accelerate the spoilage rate of pork jerky. It may also be due to the water retention of meat reduced at 37°C, resulting in a large loss of water (Gao, 2019). The decrease rate of the Aw of the blank group was greater than the experimental group. Although a lower Aw can inhibit the growth of most of the microorganisms in pork jerky, most molds and other microorganisms can survive under lower Aw conditions, resulting in a shorter storage period in the blank group (Ling et al., 2019). However, the pork jerky added with ε‐PL, due to the broad antibacterial effect of ε‐PL, made it difficult for most microorganisms to grow and reproduce under low Aw conditions, which greatly delayed the rate of spoilage of pork jerky, and the storage period of the pork with ε‐PL added was extended effectively (Juneja et al., 2016).

### 3.5 Comparison of the effects of ε‐PL and common preservatives

Meat jerky contains a lot of nutrients, and it was easy to breed microorganisms and oxidize during storage, which will cause the meat jerky to spoilage. In this context, researchers often use sorbate, D‐isoascorbate, sodium nitrite, and other preservatives or antioxidants in the production process of meat jerky, to ensure the quality of meat jerky and extend the storage period of meat jerky.

In this experiment, the microbiological test was carried out on the pork jerky with ε‐PL on the 180th day. The test results showed that the aerobic plate count in the pork jerky was 800 CFU/g (27℃) and 1,300 CFU/g (37℃) on the 180th day (not shown in table or figure), conforming to national standards. At the same time, the preservatives or antioxidants which used in different varieties of commercially available meat jerky were collected, and the storage period of the different varieties of meat jerky products was compared with the data obtained in this experiment, to verify the application potential of ε‐PL as a natural preservative in meat jerky products. The comparison results were shown in Table 2. (The brand of commercial jerky was replaced by letters.)

**TABLE 2:** Comparison of the antiseptic effects of ε‐PL and commercial preservatives or antioxidants

The product name	Preservatives or antioxidants	Storage time (days)
This experiment		
A	ε‐PL	At least 180
Beef jerky		
A	Sodium nitrite	150
B	Nisin, Sodium dehydroacetate	180
C	Potassium sorbate	180
D	Potassium lactate, D‐isoascorbate, Sodium nitrite	360
Pork jerky		
A	Potassium sorbate	120
B	D‐isoascorbate, Glycerin, Sodium lactate	180
C	Potassium sorbate, Nisin, Sodium dehydroacetate, Sodium lactate	360
Fish jerky		
A	Potassium sorbate	120
B	Potassium sorbate	180
C	Potassium sorbate, Citric acid	270
Duck jerky		
A	D‐isoascorbate, Nisin	270
B	Potassium sorbate	300
Chicken jerky		
A	D‐isoascorbate, Sodium dehydroacetate	180
B	D‐isoascorbate, Potassium sorbate, Sodium nitrite	180

It can be seen from Table 2 that the preservatives or antioxidants of different kinds of meat jerky on the market that mostly use potassium sorbate, sodium nitrite, sodium dehydroacetate, and D‐isoascorbate, which has been used maturely, may be effective on extending the storage period of products. However, these chemicals inevitably cause harm to human body and affect human health. In this experiment, the ε‐PL was used in pork jerky, and the storage period could reach at least 180 days, which was comparable to many meat jerky products with chemical preservatives or antioxidants. This experiment provides a reference for the industrial application of ε‐PL.

### 3.6 Application prospect of ε‐PL in meat jerky products

This experiment found that ε‐PL was used as a natural preservative in pork jerky, and the preservation effect was significantly better than the blank group. During the storage process, due to the broad antibacterial spectrum of ε‐PL, it had a good inhibitory effect on various microorganisms such as *Coliforms*, *Staphylococcus aureus*, *Salmonella*, and *Shigella*. In addition, ε‐PL was easily soluble in water, so that the pork jerky could evenly absorb ε‐PL during the soaking process, which improved the preservation effect. Moreover, ε‐PL was decomposed into L‐lysine in the body, which was beneficial to human health. Therefore, ε‐PL is a natural, nontoxic, and safe preservative. Furthermore, the consumption of ε‐PL is only 0.25 g/kg meat, and thus the cost is small, so it is suitable for actual production (Zhu et al., 2020).

In this experiment, it is based on the traditional processing technology of pork jerky and explored the role of ε‐PL in extending the storage period of only spiced pork jerky. It is necessary to further research the market demand to produce spicy flavor, barbecue flavor, and other flavors of pork jerky to satisfy consumer tastes. In addition, ε‐PL can also be applied to common meat jerky products such as beef jerky, duck jerky, and chicken jerky to satisfy consumers' pursuit of natural and healthy food.

## 4 CONCLUSION

In this experiment, during the machining processing of pork jerky, it was divided into experimental group and blank group based on whether the pork jerky was added ε‐PL. The test results found that the *Staphylococcus aureus* was detected in the blank group (27℃) when 18 days which the storage period was thus 15 days, and the aerobic plate count was 2,100 CFU/g at 15 days; the *Staphylococcus aureus* was detected in the blank group (37℃) when 12 days which the storage period was thus 9 days, and the aerobic plate count was 3,300 CFU/g at 9 days. Due to the detection of pathogenic bacteria, the blank groups were judged as expired, and subsequent test was stopped. On the 60th day, the aerobic plate count of experimental group (27℃) was 120 CFU/g, and the aerobic plate count of experimental group (37℃) was 150 CFU/g, while *Coliforms*, *Staphylococcus aureus*, *Salmonella*, and *Shigella* were not detected in the experimental groups. The test results show that the pork jerky with ε‐PL conforms to the Chinese national standard for the regulation of microorganisms in meat jerky which means the aerobic plate count is ≤10,000 CFU/g, *Coliforms* are ≤10 CFU/g, and *Staphylococcus aureus*, *Salmonella*, and *Shigella* must not be detected, which indicates that ε‐PL has inhibitory effect on the growth of microorganisms in pork jerky. The TVB‐N content of pork jerky in the blank group reached 14.00 mg/100 g (15th day, 27°C) and 14.93 mg/100 g (9th day, 37°C) at the end of the storage period. On the 60th day, the TVB‐N content of the experimental group was 11.20 mg/100 g (27℃) and 15.86 mg/100 g (37℃), which indicates that ε‐PL effectively inhibits the growth of microorganisms and has a good microbial stability. In addition, the pH and Aw of the experimental group and the blank group both decreased during the entire storage process, but the decrease rate of the blank group was greater than the experimental group, indicating that ε‐PL can stabilize the quality of pork jerky. Finally, the antiseptic effect of ε‐PL was compared with the common preservatives or antioxidants of different kinds of jerky on the market. It was found that ε‐PL was comparable to chemical preservatives such as potassium sorbate and sodium nitrite to a certain extent. Therefore, as a natural preservative that is beneficial to human health, ε‐PL has high research value in the storage of meat products such as meat jerky.

## CONFLICT OF INTEREST

The authors declare that they have no conflict of interest.

## AUTHOR CONTRIBUTION

**Yizhuo Zhang:** Conceptualization (lead); Formal analysis (lead); Software (lead); Writing‐original draft (lead); Writing‐review & editing (equal). **Changqing Zhao:** Data curation (equal); Funding acquisition (lead); Resources (lead); Supervision (lead); Writing‐review & editing (equal). **Xingxiu Zhao:** Methodology (equal); Project administration (equal). **Yiguo He:** Formal analysis (equal); Methodology (supporting); Resources (equal).

## ETHICAL APPROVAL

The study did not involve any human or animal testing.

## INFORMED CONSENT

Written informed consent was obtained from all study participants.

## Data Availability

The data that support the findings of this study are available from the corresponding author upon reasonable request.
